# Diagnosis of cutaneous sarcoidosis; clinical and the prognostic significance of skin lesions

**DOI:** 10.1186/2049-6958-8-26

**Published:** 2013-03-22

**Authors:** Halil Yanardag, Cuneyt Tetikkurt, Muammer Bilir, Sabriye Demirci, Aydin Iscimen

**Affiliations:** 1 Department of Internal Medicine, Cerrahpasa Medical Faculty, Istanbul University, Istanbul, Turkey; 2 Department of Pulmonary Medicine, Cerrahpasa Medical Faculty, Istanbul University, Istanbul, Turkey; 3 Department of Dermatology, Cerrahpasa Medical Faculty, Istanbul University, Istanbul, Turkey

**Keywords:** Sarcoidosis, Cutaneous sarcoidosis, Erythema nodosum, Lupus pernio, Skin plaque, Punch biopsy, Sarcoidosis, Skin plaque

## Abstract

**Background:**

Sarcoidosis is a systemic disease characterized by the formation of noncaseating granulomas in various tissues. Cutaneous involvement occurs in 20 to 35 percent of the patients and may be the initial manifestation of the disease. Our study was performed to discriminate the clinical, laboratory, and prognostic differences between patients with specific and nonspecific cutaneous involvement. The second aim was to asses the diagnostic usefulness of punch biopsy in sarcoidosis.

**Methods:**

The clinical, laboratory, pathological features, and skin biopsy results of 120 patients with cutaneous sarcoidosis were evaluated. The patients fulfilled clinical, radiologic or both features of sarcoidosis supported by the histopathologic evidence of noncaseating granulomas.

Skin involvement was the initial finding in 30% of the patients. Erythema nodosum and lupus pernio were the most common skin lesions. Almost all of the patients with LP were either stage 0 or 1. Respiratory symptoms occurred in 72.2% of the patients with specific skin involvement. BronchoalveolarLavage (BAL) lymphocytosis, high ratio of CD_4_/CD_8_ and elevated serum Angiotensin Converting Enzyme (ACE) were more frequent in patients with specific cutaneous lesions. The frequency of progressive disease was significantly higher in this group. Punch skin biopsy was diagnostic in 81.6% of the patients with a complication rate of 4%.

**Conclusions:**

Specific cutaneous lesions along with BAL lymphocytosis, high CD_4_/CD_8_ ratio and elevated serum ACE levels may be predictors of progressive disease in sarcoidosis. Punch biopsy is a simple technique with a high diagnostic yield and a low complication rate for cutaneous sarcoidosis.

## Background

Sarcoidosis is a multi-system disease of unknown etiology that has a wide variety of clinical manifestations and frequently, an unpredictable course. It involves mainly the lungs, mediastinal and peripheral lymph nodes, skin, liver, spleen, eyes and parotid glands. Less frequently, but usually severe, manifestations also occur in the central nervous system, heart, upper respiratory tract and bones. Because lesions can exhibit many different morphologies, cutaneous sarcoidosis is known as one of the great imitators in dermatology [1-3].

The prevalence of a particular type of cutaneous lesion varies among races as well as individual cases. The frequency of specific cutaneous involvement in sarcoidosis ranges from 9% to 37% [4,5]. Although different series report variable incidences of skin involvement during the course of the disease, nearly a quarter of sarcoidosis patients have occurrence of skin lesions [6,7]. Cutaneous involvement in sarcoidosis may occur at any stage of the disease but most often present at the onset and the patients are often seen initially by a dermatologist [2,3]. Different skin lesions associated with sarcoidosis have been reported. They are divided into two groups as: specific skin lesions where histological examination shows the typical sarcoid granulomas, and non-specific skin lesions. Specific lesions are lupus pernio (LP), infiltrated plaques, maculopapular eruptions, subcutaneous nodules and scars, and rare morphologies such as alopecia, ulcers, hypopigmented patches, and ichthyosis. Despite the clinical importance of scar infiltrates in the diagnosis of sarcoidosis, it is often overlooked because the lesions are usually small and asymptomatic. Erythema nodosum is the most frequent nonspecific skin lesion in sarcoidosis [2,6,7].

We retrospectively analyzed the clinical and dermatological features of sarcoidosis at our center. The aim of our study was to determine the correlation between specific or nonspecific skin lesions, involvement of other organs, clinical findings, disease stages and to establish the diagnostic usefulness of punch biopsy for cutaneous sarcoidosis.

## Methods

One hundred and twenty patients attending our center between September 1966 and January 2011 were included in the study. The patients fulfilled clinical, radiologic or both features of sarcoidosis supported by the histopathologic evidence of noncaseating granulomas. The skin changes reported by the patients and noted in the medical records were taken into account. All patients had specific or nonspecific skin lesions confirmed by punch, or wedge biopsy. The medical charts were used to obtain data about patient’s age, sex, radiologic stage, laboratory findings, pulmonary function tests, bronchoalveolar lavage (BAL) and histopathologic results. Sputum or bronchial lavage culture was done to exclude infection.

Central nervous system involvement was considered to exist if a lesion was confirmed by computed tomography (CT) or magnetic resonance imaging (MRI) and diagnosed by a consultant neurologist. For evidence of ocular sarcoidosis all patients were screened by an opthalmologist. Abnormal liver function tests, hypercalcemia and hypercalcuria were considered to be present if they were above the normal range. Spirometry was performed according to the ATS/ERS recommendations with a body plethysmograph unit (Zan 500, Messgeraete, Oberthulba, Germany). DL_CO_ was measured with the single-breath technique and was adjusted for alveolar ventilation (V_A_). The evidence of restrictive (reduced TLC or FVC and normal or high FEV_1_/FVC), obstructive disease (FEV_1_/FVC <70%) or decreased diffusion capacity (DL_CO_/VA <80%) were considered abnormal. All patients had tuberculin test, chest x-ray and punch or wedge biopsy of the skin. Chest roentgenograms were staged as follows: stage 0: normal, stage 1: bilateral hilar lymphadenopathy (BHL), stage 2: BHL and parenchymal involvement, stage 3: parenchymal involvement only and stage 4: pulmonary fibrosis [8]. Serum Angiotensin Converting Enzyme (ACE), chest CT and BAL were performed in 74 (61.6%), 71 (58.3%) and 54 (45%) of the patients, respectively.

For the bronchoalveolar lavage, the fiberoptic bronchoscope was wedged into a subsegmental bronchus of the middle lobe or lingula after routine inspection of the tracheobronchial tree. Prewarmed 0.9% saline solution to 37°C in five 20 ml aliquots up to a total volume of 100 ml was infused into the subsegmental bronchus and at least 40 ml or 40% of the infused volume was recovered back. After mucus removal BAL sample was cytocentrifugated for examination. The slides were stained with Giemsa or hematoxylin-eosin. A differential cell count was performed on at least 300 non-epithelial cells.

Skin punch biopsy was done on the site with the most advanced inflammatory changes. Isopropyl alcohol or providone-iodine was used as a skin antiseptic to prepare the biopsy site. Lidocaine 2% was applied for local anesthesia. Biopsies were performed with disposable knives of 4–6 mm in diameter. Punches no more than 5 mm were done for biopsy. Incisional wedge biopsy was performed in patients with nondiagnostic punch biopsy results. The histopathologic changes in specific skin lesions consisted of dermal nests, clusters of noncaseating epithelioid granulomas with minimal inflammatory cells, and variable giant cells. Necrobiotic collagen and necrosis were absent with sparse lymphocytic infiltrate concentrated peripherally around the noncaseating granulomas.

Thirty-two (26.6%) patients were treated with methylprednisolone 0.25-0.50 mg/kg for at least 12 months. Azathioprine 2 mg/kg was commenced in 9 (7.5%) patients unresponsive to steroid treatment.

For statistical analysis of the data, chi-square and student’s t test were used. Correlation between radiologic involvement, serum ACE and pulmonary function was determined by the application of Spearman rank test. A p value less than 0.05 was considered as significant.

## Results

All patients presented with various skin lesions at admission or during the course of the disease. Of the 120 cases 82 (68.3%) were women. The age ranged between 18 and 56 years and the mean age was 38.4 ± 11.6 years. The distribution of skin lesions and the dermographic features of the patients are shown in Table 1. Erythema nodosum (EN) followed by maculopapular lesions was the most frequent skin lesion. Comparison of clinical and laboratory findings in regard to skin involvement is shown in Table 2. Out of the 41 patients with abnormal pulmonary function test results, 24 (20%) had restrictive, 10 (8%) had mixed and 7 (5.8%) had obstructive type of pulmonary function abnormality. Eighteen patients (15%) had diffusion impairment.

**Table 1:** The frequency of skin lesions and the dermographic features

**Type of lesion**	**N. of patients (%)**	**Age (mean ± SD)**	**N. (%) of female patients**
Erythema nodosum	25 (20.8%)	38.6 ± 8.4	19 (15.8%)
Lupus Pernio	21 (17.5%)	41.8 ± 9.2	12 (10%)
Maculopapular lesions	19 (15.8%)	40.3 ± 7.1	14 (11.6%)
Plaque	14 (11.6%)	42.6 ± 6.2	9 (7.5%)
Nodules	10 (8.3%)	36.9 ± 10.4	6 (5%)
Scar	8 (6.6%)	39.4 ± 5.3	7 (5.8%)
Verrucose outgrowths	7 (5.8%)	44.9 ± 14.1	5 (4.1%)
Subcutaneous lesions	6 (5%)	37.4 ± 11.2	3 (2.5%)
Ulcerative and vesicular lesions	5 (4.1%)	42.6 ± 8.3	3 (2.5%)
Ichthyosis	3 (2.5%)	39.4 ± 3.9	2 (1.6%)
Erythema multiforme	2 (1.6%)	38 ± 5.6	2 (1.6%)

**Table 2:** Comparison of clinical and laboratory findings

	**Clinical profile of 72 patients with specific skin involvement (n, %)**	**Clinical profile of 48 patients with nonspecific skin involvement (n, %)**	**Statistical significance**
Respiratory symptoms	50 (69.4%)	30 (62.5%)	NS
Eye involvement	31 (43.0%)	17 (35.4%)	NS
Central nervous system involvement	7 (9.7%)	3 (6.2%)	NS
Abnormal pulmonary function test	41 (56.9%)	26 (54.1%)	NS
Abnormal liver function test	17 (23.6%)	10 (20.8%)	NS
Hypercalcemia	8 (11.1%)	5 (10.4%)	NS
Hypercalciuria	12 (16.6%)	7 (14.5%)	NS
Elevated serum ACE level	42 (58.3%)	14 (29.1%)	r = 0.76, p < 0.05
BAL lymphocytosis (>28%)	30 (41.6%)	11 (22.9%)	r = 0.72, p < 0.01
BAL CD_4_/CD_8_ ratio (> 3.5)	28 (38.8%)	10 (20.8%)	r = 0.70, p < 0.05

Chi square and Spearman rank correlation tests were used for the statistical analysis of data.

NS, not statistically significant at p < 0.05.

Punch biopsy was diagnostic in 98 (81.6%) patients and incisional wedge biopsy was done in the remaining ones to identify skin involvement. Eight patients reported a minimal sense of pain during the procedure. The major complications of punch biopsy were bleeding and infection which occurred in 5 (4.1%) and 3 (2.5%) patients respectively. The total complication rate was 4%. Seventy-two patients (60%) had specific skin lesions and they occurred more frequently in stages 2 and 3 while nonspecific lesions were more common in stage 0 and 1 (p < 0.001). In thirty-six (30%) patients, skin involvement was the initial finding of sarcoidosis. Of these patients, 12 had BAL lymphocytosis, 9 had high CD_4_/CD_8_ ratio, 10 had elevated serum ACE levels, and 24 had specific cutaneous involvement (p < 0.05). Inflammatory markers were normal in eight patients. Fifty-four patients (45%) had dry, cough, dyspnea and chest discomfort while 32 patients (26.6%) had systemic symptoms like fever, fatigue, malaise, arthralgia and the remaining 16 patients (13.3%) had specific organ related symptoms.

Out of the 74 patients with specific skin lesions, 47 63.5% had systemic involvement. Systemic disease occurred in 29 patients (60.4%) with nonspecific skin involvement. Although respiratory symptoms and other organ involvement were less frequent in patients with nonspecific skin lesions there was no significant difference (p < 0.32) between the two groups in terms of systemic disease. High CD_4_/CD_8_ ratio, high intensity alveolitis (T-cell percentage > 28%) and elevated serum ACE levels were significantly more common in patients with specific cutaneous involvement (p < 0.01) (Table 2). BAL lymphocytosis and high CD_4_/CD_8_ ratio correlated with high serum ACE levels (r = 0.64, r = 0.76, p < 0.001). In stage 0 patients with skin lesions as the initial finding, six had BAL lymphocytosis, four had high CD_4_/CD_8_ ratio, and three had abnormal ACE levels (p < 0.32). Levels of liver function test, serum and urinary calcium were not significantly different between patients with specific and nonspecific cutaneous involvement (p < 0.46) (Table 2). Tuberculin test was negative in 97 (80.8%) patients. Chest roentgenogram analysis revealed a statistically significant trend among patients with specific skin lesions (p < 0.01) in regard to disease stage (Table 3). The most frequent CT findings were widespread nodules distributed among the bronchovascular bundles or subpleurally followed by interstititial infiltrates, hilar and mediastinal adenopathy, consolidation and architectural distortion with fibrotic changes of the lung parenchyma. The correlation between widespread nodules and the specific lesions was high (r = 0.82, p < 0.05). Interstitital infiltrates were more common in patients with nonspecific cutaneous lesions (r = 0.71, p < 0.05).

**Table 3:** Distribution of patients with specific and nonspecific cutaneous involvement according to the stage of sarcoidosis

**Stage**	**N. of patients with specific skin involvement (%)**	**N. of patients with nonspecific skin involvement (%)**
0	7 (5.8%)	9 (7.5%)
I	28 (23.3%)	22 (18.3%)
II	21 (17.5%)	10 (8.3%)
III	12 (10%)	4 (3.3%)
IV	6 (5%)	1 (0.83%)

The features for progressive disease were manifestation of new systemic or organ related symptoms, progression of previous symptoms, deterioration of pulmonary function tests and/or radiologic findings. The frequency of progressive disease was more significant (p < 0.05) in patients with specific cutaneous lesions (Table 4). Patients with erythema nodosum had the best prognosis (p < 0.001). There was no correlation between BAL lymphocytosis or high CD_4_/CD_8_ ratio and pulmonary function test results and DL_CO_ (r = 0.24, p < 0.05; r = 0.18, p < 0.05).

**Table 4:** Disease course and type of skin lesions

	**N. of patients stable or improved (%)**	**N. of patients with progressive disease (%)**	**Statistical significance**
Patients with specific skin involvement	46 (62.1%)	28 (37.8%)	p < 0.01
Patients with nonspecific skin involvement	41 (89.1%)	5 (10.8%)	p < 0.01

The mean follow up time was 11.8 years. Fifty-eight percent of stage I patients had spontaneous resolution while the remaining ones progressed to stage II. Forty eight percent of these patients progressed to stage III. In stage III, 58% resolved while 18% progressed to stage IV disease. Following immunosuppressive treatment, the elevated serum ACE levels decreased significantly (p < 0.05) in patients with specific cutaneous lesions. In patients with nonspecific skin involvement, correlation of treatment and serum ACE decrease was not significant. Mortality rate was 15% (18 patients) and in 10 patients it was due to causes other than sarcoidosis. Four patients died of respiratory insufficiency and 5 patients died of sudden heart attack.

## Discussion

Sarcoidosis is a systemic granulomatous disease of unknown origin that can affect any organ in the body. The lungs, eyes, skin, liver, and lymphatic system are most commonly involved. Skin lesions may appear at any stage of the disease and specific skin lesions may occur in 9% to 37% of the patients [4,5]. Lesions often appear at the onset of systemic illness, providing a valuable opportunity for early diagnosis [9,10]. Cutaneous sarcoidosis may develop with or without systemic involvement. Because the skin lesions may present with a wide spectrum of different morphologies, sarcoidosis becomes a diagnostic challenge for the internist, especially when the lesions are nonspecific or when there is no systemic involvement [11,12]. The purpose of our study was to determine the association between specific or nonspecific skin lesions and involvement of other organ systems, laboratory findings, disease course, and to demonstrate the diagnostic yield of punch biopsy in cutaneous sarcoidosis.

Cutaneous sarcoidosis was the initial manifestation in approximately one third of our patients and was a compatible finding with the literature data [4,5]. BAL lymphocytosis, high CD_4_/CD_8_ ratio, and elevated serum ACE levels in this group may be considered as important laboratory markers for the diagnosis of sarcoidosis. Although not statistically significant, BAL lymphocytosis, particularly with a high CD_4_/CD_8_ ratio, and an elevated serum ACE, were highly suggestive of sarcoidosis in the context of nonspecific cutaneous involvement with no other systemic findings. The decreased frequency of respiratory symptoms, systemic involvement and lack of laboratory abnormalities were more common in patients with erythema nodosum. On the other hand, BAL lymphocytosis, high CD_4_/CD_8_ ratio, and elevated serum ACE levels were significantly more frequent in patients with specific skin lesions. These laboratory findings may be associated with the granulomatous burden of the specific skin lesions. Activated T cells and macrophages in specific cutaneous lesions release a number of cytokines that cause progressive disease.

Skin involvement in females were approximately two times more than males. This is probably due to estrogen that may play a role in the development of skin lesions [13]. Another factor is the failure of every patient to undergo a routine dermatologic examination. This may be explained by the possibility that females have a greater tendency than males to observe their body or skin for these type of lesions.

There was no significant difference in the frequency of laboratory abnormalities, except the high frequency of negative tuberculin test in patients with specific skin lesions. The presence of abnormal pulmonary function tests, ocular, and neurological involvement was not significantly different between patients with specific and nonspecific cutaneous involvement but was more common in the former. The trend toward radiologically more advanced disease was more frequent in patients with specific skin disease along with BAL lymphocytosis, high CD_4_/CD_8_ ratio and an elevated serum ACE level. Definitive correlations between specific histopathologic features and clinical disease have not been demonstrated to date. Hanno and Mana have reported that specific skin lesions usually have no prognostic significance, do not show any correlation with the extent of systemic involvement and do not indicate a more serious form of sarcoidosis [9,12]. Our findings contradict the results of these studies, but the the sample size of our study is much greater than that in the others. However, Olive has noted that patients with skin lesions are more likely to have systemic involvement which supports our results [4]. The frequency of cutaneous involvement and particular types of skin lesions of sarcoidosis vary across different races or regions [14,15]. In addition to sarcoidosis being more frequent in individuals of Afro-American origin, skin involvement in these individuals may also have a more severe form [16]. Ethnic differences may play an important role for skin and systemic involvement as well as progressive disease. Our patient population included had a very wide ethnic base and it was almost impossible to identify the ethnic origin. They were followed up at least for five years after the initial diagnosis and are currently under control. The minimum six years follow-up is a sufficient period of time for a medical prognosis of sarcoidosis in regard to cutaneous involvement. Our findings are statistically significant but they may not be clinically significant. Further studies with larger populations are needed to discriminate clinical and prognostic differences between patients with specific and nonspecific cutaneous lesions in sarcoidosis.

A recognition of cutaneous lesions is important because they provide a visible clue to diagnosis especially if they have a specific appearance. These lesions also are an easily accessible source of tissue for diagnosis. Punch biopsy provided sufficient tissue for histopathologic examination, was diagnostic of both sarcodosis and sarcoidosis associated cutaneous lesions in most of our patients. In the remaining patients, histopathological examination of the incisional wedge biopsy confirmed the diagnosis. We believe that punch biopsy is a simple technique to learn and it can easily be performed for cutaneous sarcoidosis with high a diagnostic yield and a low rate of complications. As the clinician becomes more experienced with the technique, the diagnostic accuracy will increase because most of the nondiagnostic punch biopsy results were the initial biopsies.

## Conclusion

In conclusion, our results suggest that the presence of noncaseating granulomas on skin biopsy along with BAL lymphocytosis, a high CD_4_/CD_8_ ratio, and an elevated serum ACE level are associated with progressive disease. Cutaneous involvement in sarcoidosis is not only useful for diagnosis but may also discriminate progressive disease. Specific lesions like lupus pernio and plaques may have a prognostic significance. Punch biopsy is a simple and accurate technique that can be easily performed for the diagnosis of sarcoidosis itself and the associated cutaneous lesions.

## Competing interest

All authors declare that they have no competing interest.
